# Fungal aneurism of the right posterior inferior cerebellar artery (PICA)

**DOI:** 10.1016/j.mmcr.2019.09.004

**Published:** 2019-09-25

**Authors:** Stefano Tambuzzi, Michele Boracchi, Francesca Maciocco, Cristina Tonello, Guendalina Gentile, Riccardo Zoja

**Affiliations:** a Laboratorio di Istopatologia Forense e Microbiologia Medico Legale, Sezione di Medicina Legale e delle Assicurazioni, Dipartimento di Scienze Biomediche per la Salute, Università degli Studi di Milano, Via Luigi Mangiagalli, 37, 20133, Milano, Italy; b Ospedale S. Carlo Borromeo, Servizio di Immunoematologia e Medicina Trasfusionale (SIMT), Via Pio II, n°3, 20153, Milano, Italy; c Pathology Unit, Luigi Sacco Hospital, ASST Fatebenefratelli Sacco, Milano, Italy

**Keywords:** Fungal aneurysm, Sudden death, Autopsy, Aspergillus, Forensic pathology

## Abstract

In this case-report, the Authors show the case of a sudden death occurred in a 38-year-old woman submitted to surgical excision of a right acoustic neurinoma. At the autopsy, was detected a cerebral hemorrhage with multifocal localization by a ruptured rare fungal aneurysm of the Posterior Inferior Cerebellar Arthery (PICA). The PCR analysis, carried out on formalin-fixed paraffin-embedded tissue, identified the *Aspergillus Penicillioides* as the involved pathogen.

We discuss the main points of infectious aneurysms, being a potential neurosurgical complication.

## Highlights

• Lethal intracranial hemorrhage by rupture of mycotic aneurysm.
• Mycotic aneurysm of PICA.
• *Aspergillus* in cadaver.

## 1 Introduction

Intracranial infectious aneurysms, although known since 1861, are scarcely reported in literature due to several factors: the rarity of these lesions, the variability in their development and clinical presentations and the lack of population-based epidemiological data [1]. They are rare cerebrovascular lesions (0,7–5,4% of all intracranial aneurysms) [2] and uncommon cause of intra-cranial hemorrhage, with severe prognosis or even lethal in 80% of cases [3]. Moreover, infectious aneurysms are often clinically silent and, for this reason, they are detected for the first time in 5–10% of autopsies [2].

These aneurysms are usually located, in 50–70% of cases, at the middle cerebral artery (MCA) or at its distal branches; less commonly at the anterior and posterior cerebral arteries or at the superior and inferior cerebellar arteries. The posterior inferior cerebellar artery (PICA) results to be involved in a low percentage of cases (0.49% - 3%) and, in this context, infectious aneurysms are typically observed deep in the posterior cranial fossa, in close anatomical relationship with the brainstem, the caudal cranial nerves and the skull base structures [4]. In particular, the main locations are placed at its origin (PICA-VA), at its distal portion and at the junction with the vertebral and basilar arteries (VA-BA). Most of infectious aneurysms involving the PICA are small size, between 5.6 and 7 mm, and they are characterized by a high propensity to bleed [5]; nevertheless, giant aneurysms are documented in literature [6].

Intracranial infectious aneurysms arise in 65% of prosthetic heart valve carriers, who have developed bacterial endocarditis, especially a left one [7], in 6.3% of drug addicts [8] and in subjects with previous intracranial bacterial infections (e.g. bacterial meningitis - 5.2%) [9], cavernous sinus thrombosis – 2.8%, orbital cellulitis, or patients that have undergone to neurosurgical procedures [10]. They are also documented in patients with dental infectious diseases, poor dental hygiene – 4.2%^3^ and in immunosuppressed subjects [11].

In the infectious aneurysms pathogenesis, an arterial wall or a preexisting intracranial aneurysm are infected by different pathogens [1], i.e. predominantly bacteria [9], less commonly mycetes [1] and exceptionally viruses [12]. These pathogens usually have an hematogenous spread, through septic emboli, in case of bacterial endocarditis, or a contiguous one from a nearby intracranial “focus” of infection [13].

In this report, we present a case of unexpected death occurred to a 38-year-old woman, due to the rupture of a fungal aneurysms involving the PICA, detected only in the post-mortem investigations. The lady had previously been subjected to acoustic neurinoma exeresis, with no evidence of preexisting aneurysms at pre-operative investigation by means of cerebral nuclear magnetic resonance (NMR) with gadolinium enhancement.

### 1.1 Case

A 38-year-old woman underwent to neurosurgical surgery consisting in the exeresis of a right acoustic neurinoma (vestibular Schwannoma) by retromastoid access. In view of good post-operative clinical conditions, the patient was discharged on the fourth post-operative day. After an initial period of well-being, the woman began to experience worsening fatigue and fever (37.8 °C), reason why she went to the A&E of the same hospital where she had been operated 15 days before. The lady died while clinical exams were still being carried out.

**Fig. 1:**
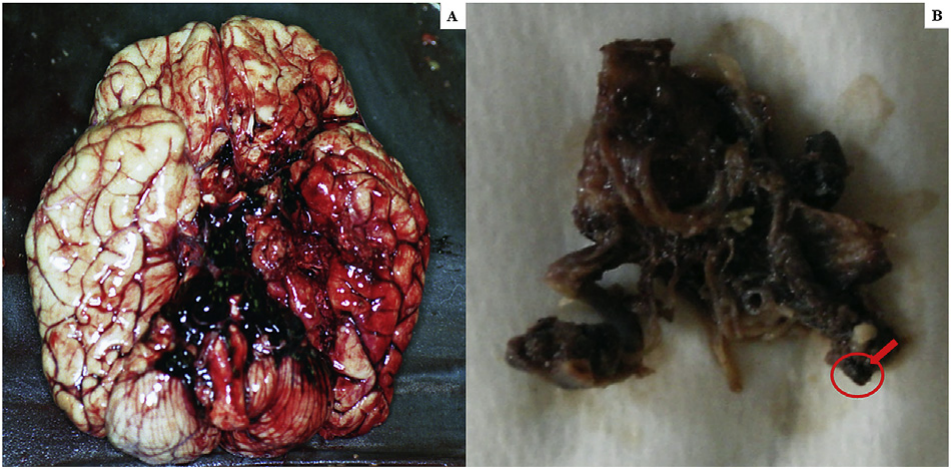

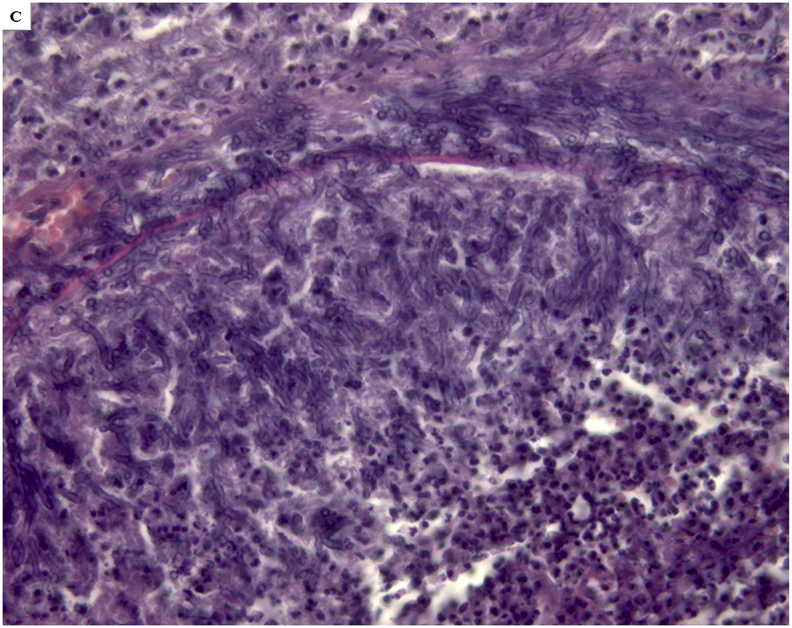

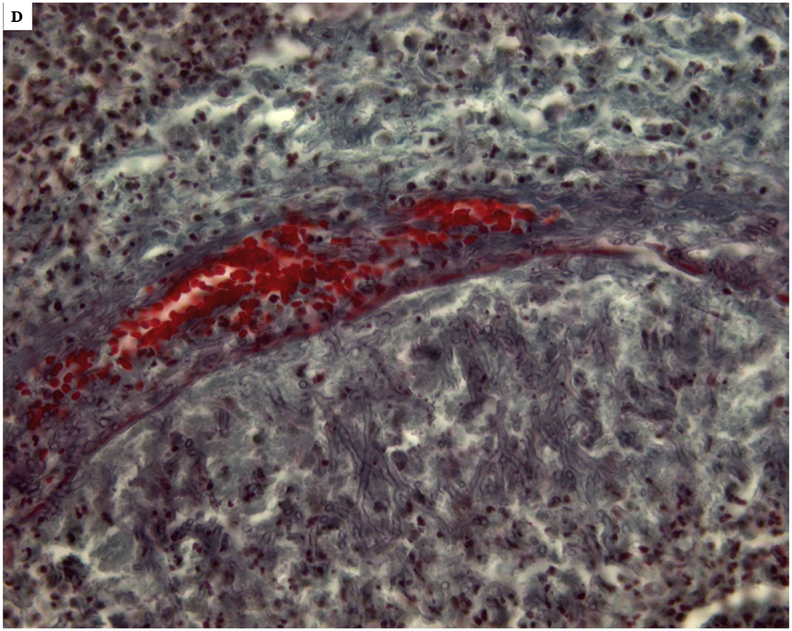

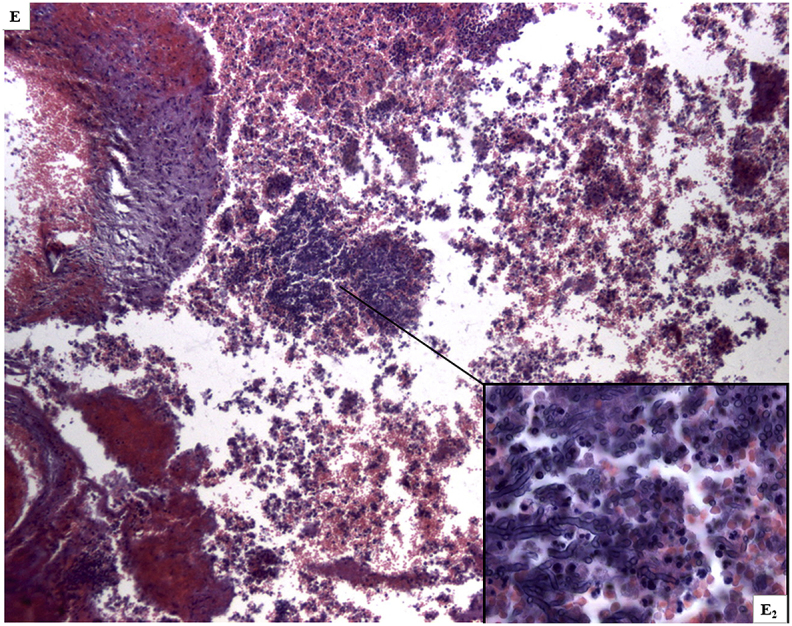

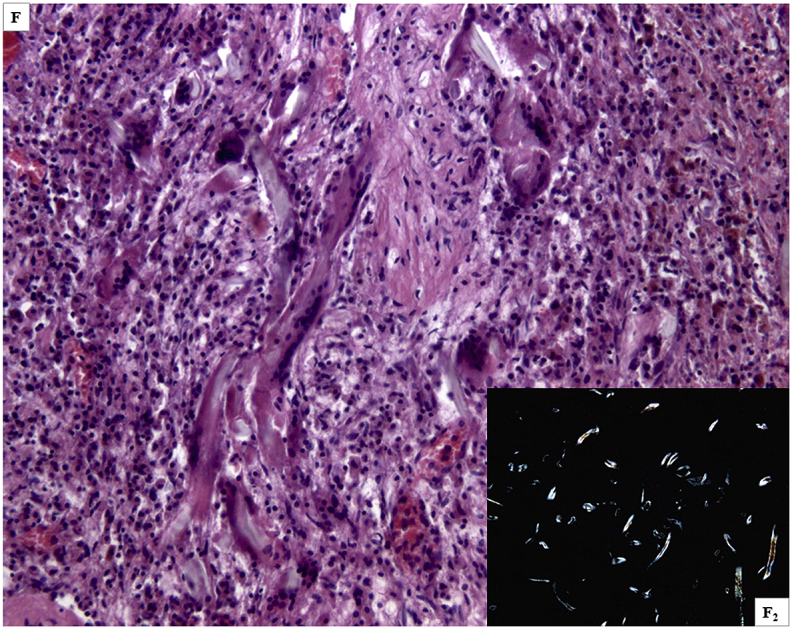
***A***. Macroscopic view of the base of the cerebral hemispheres with massive hemorrhage in correspondence of the clivus; ***B*** – Middle-posterior portion of the isolated circle of Willis with rupture of the right PICA aneurysm (arrow) in the context of a marked peri-aneurismatic hemorrhagic infiltration; **C**. Microscopic view of the PICA wall with the fungal aneurysm, assessed with H&E, 400x; ***D***. Microscopic view of septate mycotic hyphae, with a dichotomous 45° bifurcation, typical for Aspergillus, in the PICA wall, assessed with Masson-Goldner's trichrome staining, (400 x); ***E***. Microscopic view of right subarachnoid cistern with fungal nidus (H&E 100x), at a higher magnification in **E**_**2**_, with Aspergillus hyphae (H&E 1000x); ***F***. Microscopic view of right subarachnoid cistern with giant-cell inflammation and macrophages characterized by the presence of hemosiderin pigments (H&E 200x) from hemostatic material, observed also in **F**_**2**_ with polarized light (200x).

The autopsy performed to clarify the cause of death, identified massive cerebral hemorrhage with multifocal localization (leptomeningeal cerebellar, peribulbar, tetraventricular, and right pericisternal) (Fig. 1A) from a breach in the context of a fusiform aneurysm of 5 mm of diameter, located 2 mm from the origin of the PICA (Fig. 1B).

**Fig. 2:**
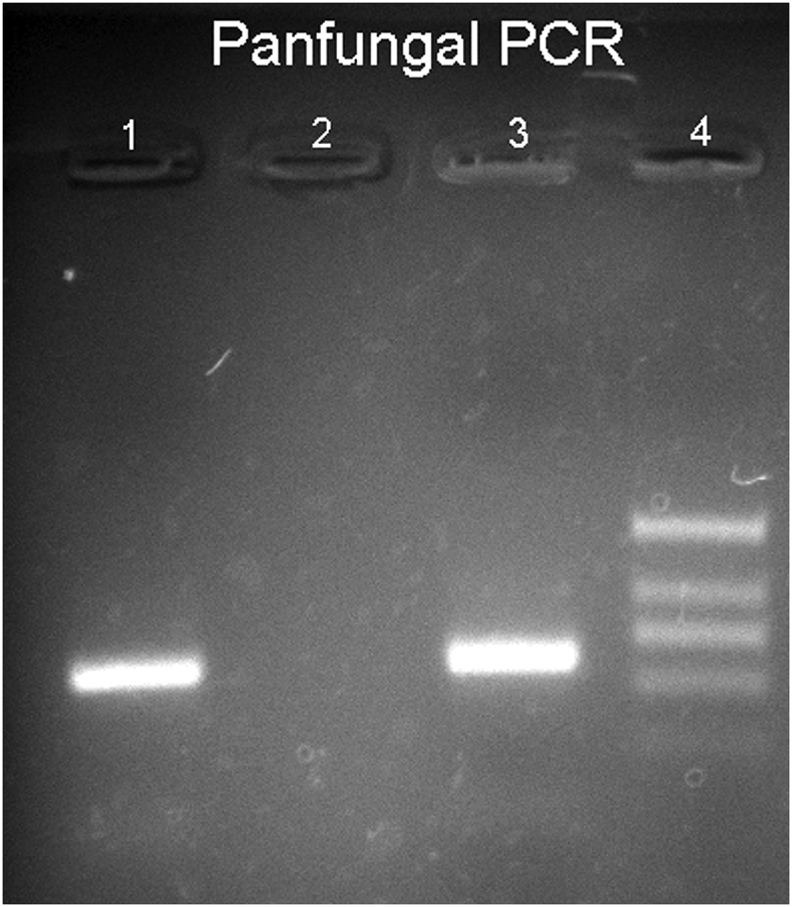
Legend to the gel electrophoresis picture: 1.Unknown sample, 2.Negative control, 3. Positive control, 4. DNA marker.

The subsequent histological examinations clarified the uncommon etiology of this aneurysm, highlighting a diffuse arterial wall colonization by septate mycotic hyphae, with a dichotomous 45° bifurcation, typical for *Aspergillus* (Fig. 1C–1F2)*.* The histological examination of all the other organs (remaining cerebral parenchyma, lungs, heart, liver, spleen and kidneys) did not show any infectious focus due to aspergillomas or invasive aspergillosis. Subsequently, we performed the molecular identification of the Aspergillus species involved. For extraction and purification of DNA from formalin-fixed paraffin-embedded tissues (FFPE) was used QIAamp DNA FFPE Tissue Kit (Qiagen). Amplification primers 5′-TCCGTAGGTGAACCTGCGG-3′ and 5′-GCTGCGTTCTTCATCGAT GC-3′ (Sigma-Aldrich) were used in a standard polymerase chain reaction (PCR) to amplify ITS1 region, located between the 18S and 5.8S rRNA genes [14]. The obtained amplicon was purified by using QIAquick PCR Purification Kit (Qiagen) and sequenced with BigDye™ Terminator v3.1 Cycle Sequencing Kit (Applied Biosystem). Reaction products were purified by 3 M Sodium Acetate Solution, pH 5.2 and ethanol precipitation, dissolved in distilled water and analyzed on 3130xl Genetic Analyzer under standard electrophoretic conditions (Fig. 2). The DNA sequence obtained was compared with that in the public DNA databases by using the BLAST interface and proved to be 97% identical to previously reported *Aspergillus Penicillioides* sequences.

Therefore, a post-mortem diagnosis of a lethal rupture of PICA fungal aneurysms was formulated. Fungal aneurysms have to be considered rare and occasional events, which are secondary to a mycotic infection.

## 2 Discussion

*Aspergillus* is a ubiquitous fungus, that can be found as a commensal in paranasal sinuses, and more than 180 species have been described [15]. Aspergillus species accounts for approximately 65% of fungal aneurysms, while the rest by Candida and Mucorale [16]. The invasiveness of *Aspergillus* is due to the production of the elastase enzyme, with which it progressively degrades the elastin present in the vessel walls, also causing the arising of inflammatory infiltrate. The development of the aneurysm is due to the proliferation of mycetes in the context of elastic lamina with its subsequent collapse and dissection from the media tunica [17]. Furthermore, the hydrostatic pressure contributes to determining the volumetric increase of the aneurysm up to its likely rupture [18].

In general, intracranial infectious aneurysms have a mainly fusiform and irregular morphology; the saccular aspect is less represented, being observed in 41% of cases [2]. These aneurysms can remain clinically silent for a long time, suddenly manifesting with non-specific signs and symptoms, i.e. headache and nausea, and with a massive intra-cranial hemorrhage, observed in 2–72% of cases [19]. Hydrocephalus may also occur. These cases, caused by the rupture of the aneurysm, have a high mortality rate of 80%; instead those in which the infectious aneurysm remains intact, lead to death in 30% of cases [1].

Currently, the diagnosis of these pathological entities is based on the documentation of an intracranial aneurysm by vascular imaging in the presence of predisposing infectious conditions. For this purpose CT scans, both with and without contrast, are extremely useful, even if the gold standard test is the conventional angiography. Classic angiographic features of infectious aneurysms are multiplicity, distal location, fusiform shape, and a change in the size or the appearance of a new aneurysm on follow-up angiogram. Finally, positive cultures from peripheral blood or the infected aneurysm wall itself can confirm the diagnosis [1].

Nowadays, the therapeutic approach of these pathological entities is not univocal and it results to be complex, due to the close topographic relationship of the neurovascular structures involved, the contiguity with the lower cranial nerves, the deep localization in the posterior cranial fossa and, finally, the high prevalence of desiccant fusiform aneurysms [4].

However, there is a wide agreement on the need for a prolonged antibiotic therapy, moving towards the selective endovascular embolization or trapping with soft and ultra-soft electrolytically detachable coils only in the cases of dynamic unruptured infectious aneurysms, carrying out repeated angiographies during the follow-up. Conventional surgical repair is restricted for ruptured aneurysms associated with hematoma and high intracranial pressure [2].

In our case, the patient developed non-specific symptoms, not immediately attributable to an intracranial aneurysm. Unfortunately, she deceased before the clinical-pathological diagnosis was performed by means of instrumental investigations. Certainly, the woman did not present preexisting aneurysms, which would have been observed at pre-operative cerebral NMR. This crucial aspect allows to place the onset of the PICA fungal aneurysm in the days after the surgery and to consider it as a rare neurosurgical complication of acoustic neurinoma exeresis. Moreover, the absence of microscopic signs of fungal infection in all of the other organs supports the hypothesis of an acute onset of this fungal aneurysm. In this case, therefore, both the topographical aspect (proximity of the aneurysm onset to the site of surgery) and temporal one (known ability of fungal aneurysms to arise in few days) [20] are coherent with the woman's clinical situation.

## Conflict of interest

There are none.
